# Effects and parameters of community-based exercise on motor symptoms in Parkinson’s disease: a meta-analysis

**DOI:** 10.1186/s12883-022-03027-z

**Published:** 2022-12-29

**Authors:** Chun-Lan Yang, Jia-Peng Huang, Ting-Ting Wang, Ying-Chao Tan, Yin Chen, Zi-Qi Zhao, Chao-Hua Qu, Yun Qu

**Affiliations:** 1 Department of Rehabilitation Medicine, West China Hospital, Sichuan University, Chengdu, 610041 Sichuan China; 2 Minda Hospital of Hubei Minzu University, Enshi, 445000 Hubei China; 3 Key Laboratory of Rehabilitation Medicine in Sichuan Province, West China Hospital, Sichuan University, Chengdu, 610041 Sichuan China; 4 Enshi Prefecture Central Hospital, Enshi, 445000 Hubei China

**Keywords:** Data synthesis, Exercise, Movement, Parkinson’s disease, Prescription, Review

## Abstract

**Background:**

Community-based exercise is a continuation and complement to inpatient rehabilitation for Parkinson's disease and does not require a professional physical therapist or equipment. The effects, parameters, and forms of each exercise are diverse, and the effect is affected by many factors. A meta-analysis was conducted to determine the effect and the best parameters for improving motor symptoms and to explore the possible factors affecting the effect of community-based exercise.

**Methods:**

We conducted a comprehensive search of six databases: PEDro, PubMed/Medline, CENTRAL, Scopus, Embase, and WOS. Studies that compared community-based exercise with usual care were included. The intervention mainly included dance, Chinese martial arts, Nordic walking, and home-based exercise. The primary outcome measure was the Unified Parkinson’s Disease Rating Scale part III (UPDRS-III) score. The mean difference (95% CI) was used to calculate the treatment outcomes of continuous outcome variables, and the I^2^ statistic was used to estimate the heterogeneity of the statistical analysis. We conducted subgroup analysis and meta-regression analysis to determine the optimal parameters and the most important influencing factors of the exercise effect.

**Results:**

Twenty-two studies that enrolled a total of 809 subjects were included in the analysis. Exercise had a positive effect on the UPDRS-III (MD = -5.83; 95% CI, -8.29 to -3.37), Timed Up and Go test (MD = -2.22; 95% CI -3.02 to -1.42), UPDRS ((MD = -7.80; 95% CI -10.98 to -6.42), 6-Minute Walk Test (MD = 68.81; 95% CI, 32.14 to 105.48), and Berg Balance Scale (MD = 4.52; 95% CI, 2.72 to 5.78) scores. However, the heterogeneity of each included study was obvious. Weekly frequency, age, and duration of treatment were all factors that potentially influenced the effect.

**Conclusions:**

This meta-analysis suggests that community-based exercise may benefit motor function in patients with PD. The most commonly used modalities of exercise were tango and tai chi, and the most common prescription was 60 min twice a week. Future studies should consider the influence of age, duration of treatment, and weekly frequency on the effect of exercise.

**PROSPERO trial registration number:**

CRD42022327162.

**Supplementary Information:**

The online version contains supplementary material available at 10.1186/s12883-022-03027-z.

## Background

Parkinson's disease (PD) is a progressive neurodegenerative disorder with both motor and nonmotor symptoms, defined by the accumulation of misfolded alpha-synuclein and the loss of dopaminergic neurons [1–5]. The number of individuals with PD increased rapidly from approximately 6.1 million in 2016 and has grown by 2.5 over the past three decades [6, 7]. By 2040, with aging and industrialization, the number of patients with PD is expected to reach a staggering 12.9 to 14.2 million [8]. Comprehensive management of PD involves pharmacology, surgical therapies, nursing, and rehabilitation [9–11]. The current treatment for PD is mainly drug therapy based on levodopa, but there are certain side effects, such as movement fluctuations, nausea, and psychosis [12, 13]. Physical therapy is the key to improving motor functions such as gait and balance that cannot be improved by drugs, so doctors should recommend it to all patients [14–17]. Rehabilitation can begin at the time of initial diagnosis and continue throughout the disease course [10, 17]. At present, rehabilitation is mainly performed with equipment or professional therapists, which improves the condition of patients but also increases the economic burden of patients. There is no evidence that PD patients are more likely to contract COVID-19 or have increased mortality [18, 19]. However, motor and nonmotor symptoms worsen in PD [19–22]. This may be because many patients and others are unable to access physical therapists or visit rehabilitation centers due to the lockdown [23]. How to continue therapy after the therapist's instruction is one of our concerns.

At present, rehabilitation research is still mainly focused on new equipment and professional techniques, ignoring economic and practical aspects. A systematic analysis of the short-term efficacy of physical therapy for PD has shown that although there are various physical therapy techniques, there is little difference in efficacy [24, 25]. Community-based exercise is a type of exercise that does not require a professional physical therapist, expensive equipment, or a particular location and is suitable for long-term recovery from illness, with easy access and low cost. Exercise is one of the most effective forms of physical therapy. Studies have also found that exercise reduces the risk of falls among older adults [26]. Animal studies have also found significant benefits in the posttraining PD model. Exercise induces the regulation of different brain regions, reduces cell death pathways, enhances neurotrophic factors and mitochondrial function, increases neurogenesis, and improves synaptic plasticity [27, 28]. People with PD engage in fewer activities of daily living and exercise than their normal peers. They need exercise to continue to consolidate the benefits. Many studies have reported the benefits of exercise at home for people with PD [29, 30]. The effects of exercise can last from 3 to 12 months [31]. In 2014, 19 physiotherapists and Parkinson's patients collaborated to develop the European Physiotherapy Guideline for PD, which described in detail the forms of exercise [32]. The main exercises included dancing, Chinese martial arts, Nordic walking, and home-based exercises. However, different studies have shown inconsistent results. The parameters vary from study to study. Currently, there has been no meta-analysis on community-based exercise, no discussion on the optimal parameters of exercise for PD, and no investigation into the factors that affect these exercises. We summarized the effects of community-based exercise on Parkinson’s motor symptoms without the help of a physical therapist or device and analyzed the appropriate parameters to provide a reference for Parkinson’s patients. This study also included a subgroup analysis and meta-regression to try to determine the main factors. This meta-analysis was conducted following the Preferred Reporting Items of Systematic Reviews and Meta-Analyses (PRISMA) statement [33] and was registered on the PROSPERO platform under the number CRD42022327162.

## Methods

### Search strategies

We performed a systematic search of the literature including studies published from inception to April 2022 in the following databases: PubMed/Medline, PEDro, CENTRAL, Scopus, Embase, and WOS. A combination of subject words and free words was adopted. The following Medical Subject Heading (MeSH) terms were included: “Parkinson’s Disease,” “exercise,” “sports,” “rehabilitation,” and “Physical Therapy Modalities.” The search clauses are listed in Supplementary Appendix S1. The language was limited to English. Additionally, we manually searched the reference list, and *Google Scholar* was searched to include more articles that met the criteria.

### Inclusion and exclusion criteria

The criteria included randomized controlled trials (RCTs) or crossover trials that compared exercise interventions with usual care for PD, regardless of disease stage and severity. The inclusion criteria are shown in Table 1.

**Table 1:** Inclusion criteria

Item	Criterion
Design	Randomized controlled trials or crossover trials
Participants	Individuals with Parkinson’s disease
Intervention	Exercise
Comparisons	Usual care
Outcome measures	Outcomes related to motor function

The exclusion criteria were as follows: 1) Exercise guided by a physiotherapist; 2) exercise therapy with equipment; 3) the control group received exercise-related therapy, including stretching or walking; and 4) the mean and standard deviation were not reported.

### Outcome measures and data extraction

Studies that assessed the effectiveness of the intervention on motor symptoms were included. The primary outcome was the Unified Parkinson’s Disease Rating Scale part III (UPDRS-III) score, which was revised to test motor function in individuals with PD in 2008 [34]. The secondary outcomes were as follows: 1) Timed Up and Go test (TUG) for a distance of 3 m; 2) the Unified Parkinson’s Disease Rating Scale (UPDRS), which assesses patients' severity, with higher scores indicating a greater severity; 3) the Berg Balance Scale (BBS); and 4) the 6-Minute Walk Test (6MWT) [32].

Endnote X20 was used to manage the literature. Data were independently extracted from eligible studies by two authors (YCL and HJP). Extracted data were compared, and any discrepancies were resolved through discussion with the third author (TYC). Relevant data, such as study time, sample size, duration of follow-up, duration of exercise, type of exercise, Hoehn and Yahr stage, and country where the studies were conducted, were extracted from all included papers. Data were collected using standard spreadsheets (Excel). If any information was unclear, we contacted the author to provide more detailed data.

### Methodological quality and data synthesis

Meta-analysis was performed using the software Review Manager (v.5.4, Nordic Cochrane Center, Copenhagen, Denmark) and Stata 12.0 (StataCorp LLC, College Station, TX, USA). Two reviewers (YCL and HJP) independently assessed the methodological quality. If the two reviewers had different opinions, we called all the participants for discussion, and finally, Professor QY made the decision. The quality of the methods was assessed using the Cochrane Collaboration risk-of-bias method quality assessment tools [35]. The variables we selected were all continuous. We extracted the means and standard deviations of the postintervention results into a predesigned Excel table. The mean difference (MD) was used as the effective value. All studies pooled effects, together with corresponding 95% CIs, to present the results of the meta-analysis. If I^2^ was < 50%, we used the fixed effects model; otherwise, we used the random effects model. We also performed subgroup analysis for the time of each treatment, frequency of treatment per week, total time of exercise per week, and duration of follow-up to analyze the optimal parameters for exercise to improve Parkinson’s motor symptoms. Finally, we also performed a meta-regression analysis to identify the factors that affected the efficacy. We selected the average age, exercise modality (dance, Chinese martial arts, others), disease stage, number of exercises per week, time of each exercise, total exercise time in the week, length of the intervention, and region of the participants (Asia, Oceania and North America) as covariates.

## Results

### Study characteristics

**Fig. 1:**
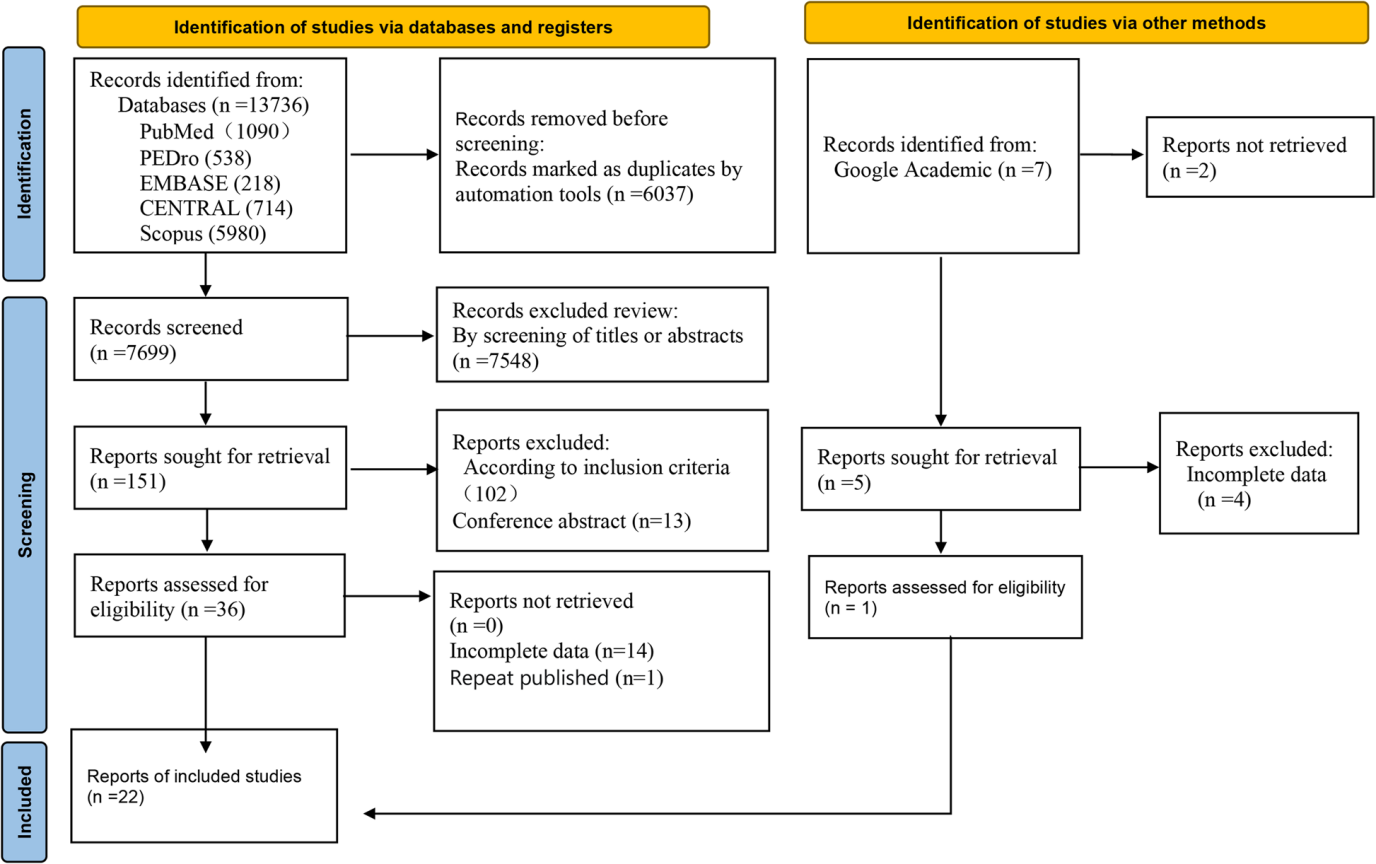
Flow diagram of the search process

The study selection process is shown in Fig. 1. A total of 151 trials that potentially met the requirements were found by reading titles and abstracts; 130 trials were excluded through intensive reading and comparison of inclusion criteria. Studies were excluded for various reasons, mainly due to the involvement of physiotherapists, incomplete data, or the use of other equipment and techniques. One study [36]reported the presence of a physiotherapist, but given that the study was outdoors and the physiotherapist was a qualified Nordic walking teacher, we included the study as well. A total of 22 full-text studies were included in the analysis [36–57]. The research information of all included studies is shown in Table 2. There were a variety of forms of physical exercise, including dance (*N* = 5), tango (*N* = 3), tai chi (*N* = 5), QiGong (*N* = 4), yoga (*N* = 2), Nordic walking (*N* = 2) and home-based exercise (*N* = 1). The studies were conducted in Asia (*N* = 9), North America (*N* = 9), Oceania (*N* = 1), and Europe (*N* = 4). Exercise interventions ranged from 4 weeks to 12 months, with 12 weeks being the dominant treatment length. The frequency of training was 1–5 times per week, but 2–3 times was the most common. Each session lasted 50, 60, or even 90 min.

**Table 2:** Characteristics of the studies

Author Year Location	Intervention	Sample (N)	Prescription of intervention	Training area	Professional instructor	Hoehn and Yahr stage	Sex (M/F)	Age	Outcome measures
Hackney 2009 USA [54]	Tango	14 (E) 17 (C)	13 weeks, 2 times/week, 60 min/session, 120 min/week	Studio	Dance instructor	1–3 (2.1)	11/3 (E) 12/5 (C)	68.2 ± 1.4 (E) 66.5 ± 2.8 (C)	UPDRS, BBS, TUG, 6MWT
Duncan 2012 USA [40]	Tango	26 (E) 26 (C)	12 weeks, 2 times/week, 60 min/session, 120 min/week	Community	Partners	1–4 (2.6)	15/11 (E) 15/11 C)	69.3 ± 1.9 (E) 69.0 ± 1.5 (C)	UPDRS-III, FOG-Q, 6MWT,
Choi 2013 S. Korea [44]	Tai Chi	11 (E) 9 (C)	12 weeks, 3 times/week, 60 min/session, 180 min/week	Clinic + community	Not mentioned	1–2 (1.6)	––	60.81 ± 7.6(E) 65.4 ± 6.8(C)	UPDRS-III, PDQ, BBS, BDI
S.-M. Cheon 2013 Korea [48]	Tai Chi	9 (E) 7 (C)	8 weeks, 3 times/week, 60 min/session, 180 min/week	Not mentioned	Instructors	1–3	––-	65.6 ± 7.9 (E) 64.9 ± 7.2 (C)	UPDRS, UPDRS-III, functional fitness
Amano 2013 USA [38]	Tai Chi	15 (E) 9 (C)	16 weeks, 3 times/week, 60 min/session, 180 min/week	Exercise studio	Not mentioned	2.4	7/8 (E) 7/2 (C)	66 ± 11 (E) 65 ± 7 (c)	UPDRS, GI, Gait
Gao 2014 China [53]	Tai Chi	37 (E) 39 (C)	12 weeks, 3 times/week, 60 min/session, 180 min/week	Community	Tai Chi instructor	1–4 (2.8)	23/14 (E) 27/12 (C)	69.54 ± 7.32 (E) 68.28 ± 8.53 (C)	BBS, UPDRS-III, TUG
Cugusi 2015 Italy [39]	Nordic Walking	10 (E) 10 (C)	12 weeks, 2 times/week, 60 min/session, 120 min/week	Not mentioned	Physical Activity professionals	1–3	2/8 (E) 2/8(C)	68.1 ± 8.7 (E) 66.6 ± 7.3 (C)	UPDRS-III, 6MWT, BBS, HY scale, BDI-II, TUG,
Rios Romenets 2015 Canada [43]	Tango	18 (E) 15 (C)	12 weeks, 2 times/week, 60 min/session, 120 min/week	Dance studio	Partner	1–3 (1.7)	12/6 (E) 7/8 (C)	63.2 ± 9.9(E) 64.3 ± 8.1(C)	UPDRS-III, TUG, PDQ39
Hashimoto 2015 Japan [41]	Dance	15 (E) 14 (C)	12 weeks, 1 time/week, 60 min/session, 60 min/week	Community halls	Video	2–4 (2.7)	3/12 (E) 7/7 (C)	67.9 ± 7.0 (E) 69.7 ± 4.0 (C)	UPDRS, BBS, TUG, SDS
Liu 2016 China [46]	Qigong	28 (E) 26 (C)	10 weeks, 5 times/week, 60 min/session, 300 min/week	Not mentioned	Not mentioned	1–3	11/17 (E) 14/12 (C)	65.84 ± 5.45 (E) 62.5 ± 3.13 (C)	TUG
Vergara-Diaz 2018 USA [57]	Tai Chi	16 (E) 16 (C)	12/24 weeks, 3 times/week, 60 min/session, 180 min/week	Hospital	Instructional DVD	1–2.5 (2.2)	9/7 (E) 7/9 (C)	65.7 ± 3.86 (E) 62 ± 7.77(C)	UPDRS-III, TUG, PDQ39
Michels 2018 USA [42]	Dance	9 (E) 4 (C)	10 weeks, 1 time/week, 60 min/session, 60 min/week	Movement studio	Dance therapist	1–4 (2.11)	–––	66.44 (E) 75.50 (C)	UPDRS-III, BBS, TUG, PDQ-39
Kunkel 2017 UK [55]	Dance	31 (E) 15 (C)	10 weeks, 2 times/week, 60 min/session, 120 min/week	Dance Centre	Dance teacher	1–3 (2.4)	19/17 (E) 6/9 (C)	71.3 ± 7.7(E) 69.7 ± 6.0(C)	TUG, 6MWT, BBS
Van Puymbroeck 2018 USA [50]	Yoga	15 (E) 12 (C)	8 weeks, 2 times/week, 60 min/session, 120 min/week	Clinic + university	Yoga therapist	1.5–3 (2.3)	10/5(E) 7/5 (C)	65.3 ± 6.09(E) 70.5 ± 4.44(C)	UPDRS, MINI, FGA, FOG
Cheung 2018 USA [52]	Yoga	10 (E) 10 (C)	12 weeks, 2 times/week, 60 min/session, 120 min/week	yoga studio	Yoga instructor	1–3 (2)	––	63.5 ± 8.5(E) 65.8 ± 6.6(C)	UPDRS-III, Oxidative status
H.J. Lee 2018 S. Korea [37]	Qi Dance	15 (E) 16 (C)	8 weeks, 2 times/week, 60 min/session, 120 min/week	Hospital	DVD + instructor	1–3 (2)	10/5 (E) 7/9 (C)	65.8 ± 7.2(E) 65.7 ± 6.4(C)	UPDRS, PDQ, BBS, BDI
Wroblewska 2019 UK [36]	Nordic Walking	20 (E) 20 (C)	12 weeks, 2 times/week, 60 min/session, 120 min/week	Outdoors	Physiotherapist^a^	2–3 (2.5)	8/12(E) 9/11(C)	72.1 ± 7.5 (E) 67.6 ± 6.6(C)	FOG-Q, TUG
P. Solla 2019 Italy [51]	Sardinian Folk Dance	10 (E) 10 (C)	12 weeks, 2 times/week, 90 min/session, 180 min/week	Not mentioned	Sardinian folk dance teacher	1–3 (2.1)	6/4 (E) 7/3 (C)	67.8 ± 5.9(E) 67.1 ± 6.3(C)	UPDRS-III, 6MWT, BBS, TUG
Kalyani 2020 Australia [49]	Dance	17 (E) 16 (C)	12 weeks, 2 times/week, 60 min/session, 120 min/week	Queensland Ballet	instructor	1–3 (1.65)	3/14 (E) 10/6 (C)	65.24 ± 11.88 (E) 66.50 ± 7.70 (C)	UPDRS III, TUG(3), BBS, FOG
P. L. Wu 2021 China [56]	Home-based exercise	49 (E) 49 (C)	4/8 weeks, 3 times/week, 30–50 min/session, 150 min/week	Home	By phone	1–2 (1.4)	26/23 (E) 30/19 (C)	65.12 ± 7.54(E) 63.65 ± 6.02(C)	UPDRS-III
Wan 2021 China [45]	Qigong	20 (E) 20 (C)	12 weeks, 4 times/week, 60 min/session, 240 min/week	Not mentioned	Qigong health coach	1–4	8/12 (E) 11/9 (C)	64.95 ± 7.83(E) 67.03 ± 7.47(C)	TUG
Li 2022 China [47]	Qigong	15 (E) 16 (C)	12 weeks, 5 times/week, 60 min/session, 300 min/week	In a particular place	Qigong health instructor	1–3	5/10 (E) 6/10 (C)	65.87 ± 6.13 (E) 63.25 ± 6.70 (C)	UPDRS-III, TUG

*Abbreviations:* *N* number, *min* minutes, *E* experimental group, *C* control group, *H&Y* Hoehn and Yahr, *UPDRS-III* Unified Parkinson’s Disease Rating Scale part III, *PFS-16* Parkinson’s Fatigue Scale, *BDI-II* Beck Depression Inventory-II, *NMSS* Nonmotor Symptoms Scale, *FU* Follow-up, *FOG-Q* Freezing of Gait Questionnaire, *PDQ39* Parkinson’s Disease Questionnaire (QoL), *UPDRS* Unified Parkinson’s Disease Rating Scale, *PDQL* the Parkinson’s Disease Quality of Life questionnaire, *BBS* the Berg Balance Scale, *BDI* the Beck Depression Inventory, *GI* Gait initiation, *TUG* Timed Up & Go test, *MINI* Mini-BESTest, *FGA* Functional Gait Assessment, *ADLs* activities of daily living, *SDS* Self-rating Depression Scale

^a^a qualified NW instructor

### Methodological quality

**Fig. 2:**
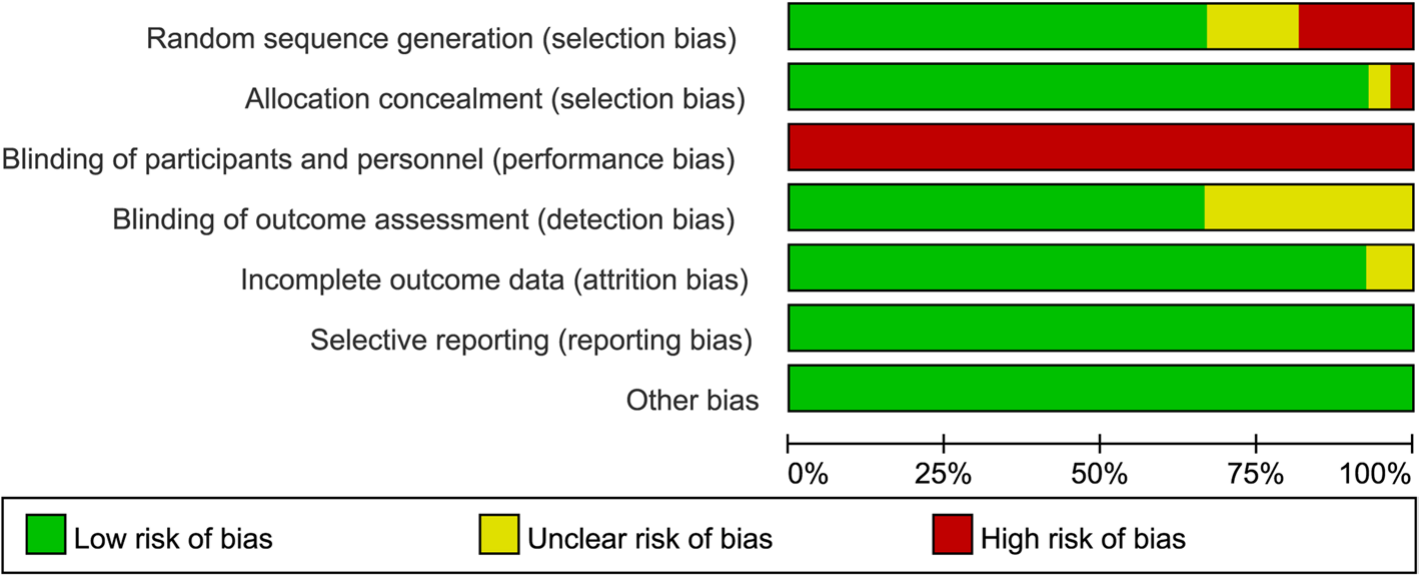
Summary of risk of bias

**Fig. 3:**
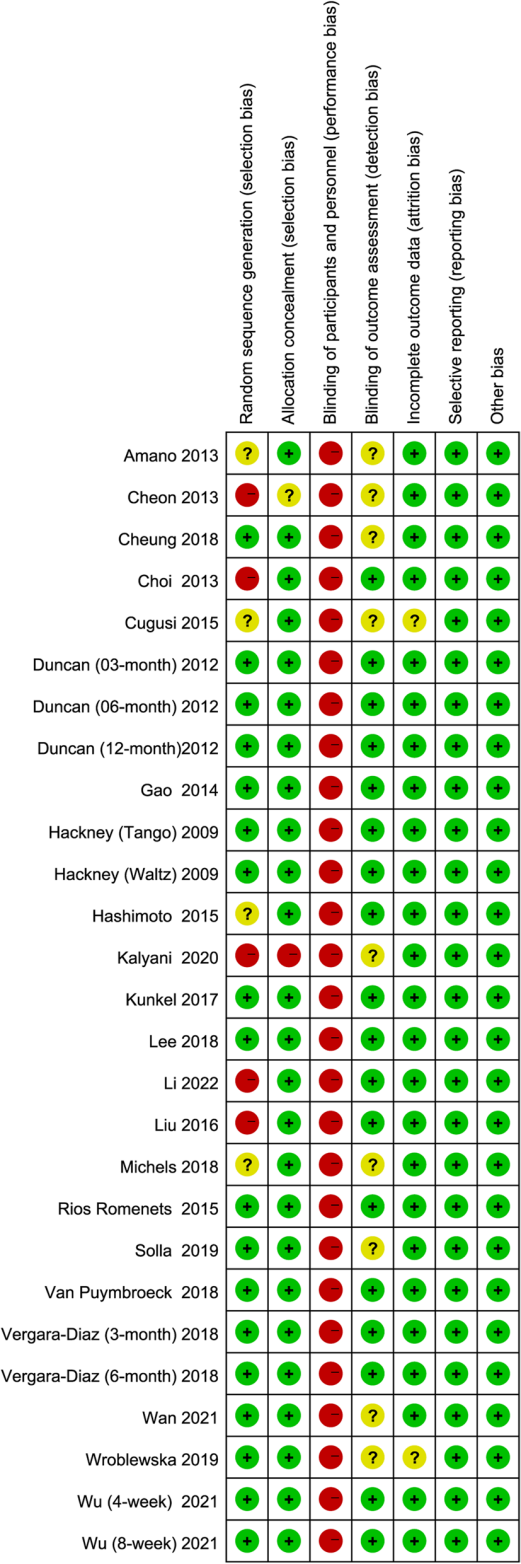
Risk characteristics of the study

The quality assessment of methods using the Cochrane Collaboration risk of bias tool showed that the methodological quality of the included trials varied widely. This was unlikely to be possible with subject blinding, and exercise outside the intervention could not be controlled. Twenty-five studies (92.59%) performed better with assignment blinding, but only 18 studies (66.67%) reported the generation of random sequences. Evaluator blinding was not reported in 9 trials (33.33%), and patients could not be blinded due to the nature of the exercise intervention. Most trials reported data loss and subjects dropping out. The risk of publication bias and other biases was low. The overall risks included in the studies and the risk assessment for each study are shown in Figs. 2 and 3.

### Effects of exercise

#### Outcome of the UPDRS-III

**Fig. 4:**
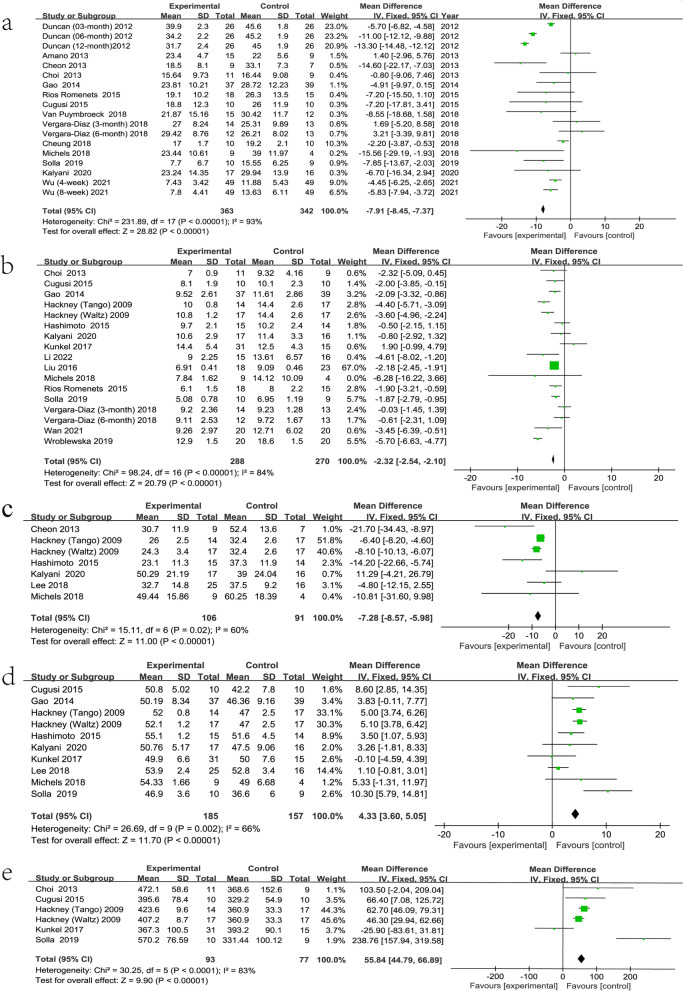
Forest plot. **a** the Unified Parkinson’s Disease Rating Scale part III (UPDRS-III); **b** the Timed Up and Go test (TUG); **c** the Unified Parkinson’s Disease Rating Scale (UPDRS); **d** the Berg Balance Scale (BBS); **e** the 6-Minute Walk Test (6MWT)

**Fig. 5:**
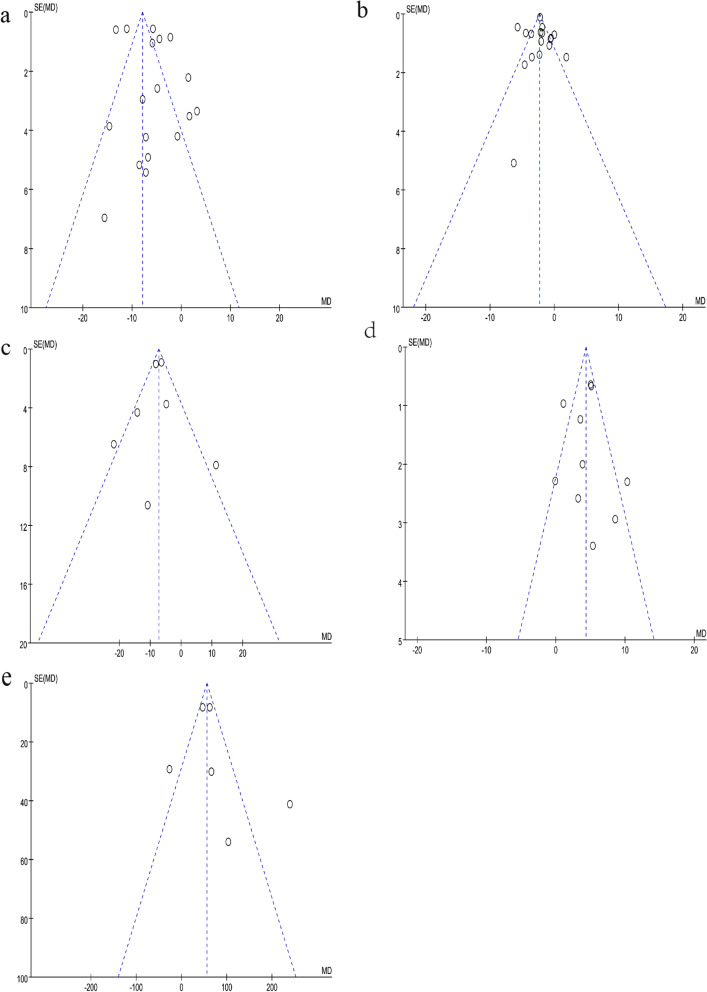
Funnel plot. **a** the Unified Parkinson’s Disease Rating Scale part III (UPDRS-III); **b** the Timed Up and Go test (TUG); **c** the Unified Parkinson’s Disease Rating Scale (UPDRS); **d** the Berg Balance Scale (BBS); **e** the 6-Minute Walk Test (6MWT)

The results of the UPDRS-III were reported in 18 of the 27 included studies. Figure 4a shows that the pooled effect estimates showed that exercise had a positive effect on UPDRS-III scores (MD = -5.83; 95% CI, -8.29 to -3.37; *P* < 0.00001). However, high heterogeneity was observed in the analysis (*P* < 0.00001; I^2^ = 93%). We did not find that eliminating one or more studies significantly changed overall heterogeneity. Two studies showed no improvement in scores, one [57] in which the exercise group scored worse than the control group before intervention and one [38] in which the scores were not statistically significant. However, there was no good reason to exclude these two studies from the analysis. From the funnel plot (Fig. 5a), eight items fell outside the 95% confidence interval. We attempted to further analyze this in a subgroup analysis.

#### Outcome of the TUG

The results of the TUG were reported in 17 of the 27 included studies. The TUG is a widely used test to assess a patient's ability to transfer. Figure 4b shows that the combined effect estimate suggested that exercise improved the TUG score (MD = -2.22; 95% CI -3.02 to -1.42; *P* < 0.00001). Figure 4b shows that high heterogeneity was observed in the analysis (*P* < 0.00001; I^2^ = 84%). According to the funnel plot (Fig. 5b), many studies fell outside the 95% CI, and no study changed the funnel plot. We attempted to further analyze this in a subgroup analysis.

#### Outcome of the UPDRS

The UPDRS can reflect the degree of PD. However, the results of the UPDRS were reported in only 6 of the 27 included studies. Figure 4c shows that the combined effect estimates indicated that exercise was beneficial in terms of the UPDRS score (MD = -7.80; 95% CI -10.98 to -6.42; *P* = 0.02). Figure 4c shows that high heterogeneity was observed in the analysis (*P* = 0.02; I^2^ = 60%). From the funnel plot (Fig. 5c), there was some bias among all the studies.

#### Outcome of the BBS

The results of the BBS were reported in 10 of the 27 included studies. Several other scales were used to assess balance function. Figure 4d shows that the pooled effect estimates indicated the beneficial effects of exercise on balance. There was a beneficial effect on the BBS scores (MD = 4.52; 95% CI, 2.72 to 5.78; *P* = 0.002). Figure 4d shows that high heterogeneity was observed in the analysis (*P* = 0.002; I^2^ = 66%). From the funnel plot (Fig. 5d), there was some bias among all the studies.

#### Outcome of the 6MWT

The results of the 6MWT were reported in 6 of the 27 included studies. Figure 4e shows that the estimated pooling effect indicated improvement in the 6MWT scores (MD = 68.81; 95% CI, 32.14 to 105.48; *P* < 0.0001). Figure 4e shows that severe heterogeneity was observed in the analysis (*P* < 0.0001, I^2^ = 83%). From the funnel plot (Fig. 5e), there was some bias among all the studies.

### Optimal parameters of exercise

We performed a subgroup analysis and detailed discussion of outcomes involving more than 10 studies. The results of the subgroup analysis of the effects of different exercise intensities on exercise are shown in Table 3.

**Table 3:** Subgroup analysis for different parameters of exercise on each motor performance measure

	UPDRS-III			TUG		
	MD (95%CI)	S	N	MD (95%CI)	S	N
**Frequency of weekly**						
1	-15.56 (-29.19, -1.93)	1	13	-2.32 (-4.00, -0.65)	2	42
2	-6.65 (-10.19, -3.11)	9	308	-0.93 (-2.53, 0.66)	7	222
3	-3.21 (-5.98, -0.44)	8	384	-5.70 (-6.63, -4.77)	3	92
4	-			-0.61 (-1.91, -0.69)	1	40
5	-			-2.04 (-3.30, -0.78)	2	62
Test for subgroup	Chi^2^ = 4.71, df = 2 (*P* = 0.09), I^2^ = 57.6%			Chi^2^ = 52.87, df = 4 (*P* < 0.00001), I^2^ = 92.4%		
**Duration of each session (min)**						
50	-5.03 (-6.40, -3.66)	2	196			
60	-5.82 (-8.75, -2.89)	15	490	-1.96 (-2.79, -1.13)	17	496
90	-7.85 (-13.67, -2.03)	1	19	-1.87 (-2.79, -0.95)	1	19
Test for subgroup	Chi^2^ = 1.01, df = 2 (*p* = 0.60), I^2^ = 0%			Chi^2^ = 0.02, df = 1 (*P* = 0.89), I^2^ = 0%		
**Duration of the week (min)**						
60	-15.56(-29.19, -1.13)	1	13	-1.22(-4.97, 2.53)	2	42
120	-7.86 (-11.51, -4.21)	8	289	-3.65 (-4.18, -3.11)	7	233
150	-4.90 (-8.57, -1.23)	2	196			
180	-6.60 (-9.06, -4.15)	7	188	-1.37 (-2.22, -0.52)	4	167
> 180mim				-2.57 (-3.71 -1.44)	3	112
Test for subgroup	Chi^2^ = 4.23, df = 3 (*P* = 0.24), I^2^ = 29.1%			Chi^2^ = 3.20, df = 4 (*P* = 0.36), I^2^ = 6.3%		

*Abbreviations: UPDRS-III* the Unified Parkinson’s Disease Rating Scale part III, *UPDRS* the Unified Parkinson’s Disease Rating Scale, *TUG* the Timed Up and Go test, *BBS* the Berg Balance Scale, *6 MWT* the 6-Minute Walk Test, *MD* mean difference, *S* the number of included studies, *N* the total number of participants, *NA* not applicable

#### Training frequency

Subgroup analysis of the frequency of weekly exercise showed a significant difference in the effect of exercise on UPDRS-III scores (*P* = 0.09, I^2^ = 57.6%) and TUG scores (*P* < 0.00001, I^2^ = 92.4%). An exercise frequency of two and three times a week had significant effects with MDs of -6.65 (95% CI: -10.19 to -3.11) and -3.21 (95% CI: -5.98 to -0.44) on UPDRS-III scores, respectively. An exercise frequency of two and three times a week had significant effects on TUG scores, with MDs of -0.93 (95% CI: -2.53 to 0.66) and -5.70 (95% CI: -6.33 to -4.77), respectively. Twice a week was the most common and effective frequency of exercise in terms of UPDRS-III scores. There was heterogeneity among subgroups in terms of TUG scores, while heterogeneity was significantly reduced when we removed the four times a week subgroup (*P* = 0.46, I^2^ = 0%).

#### Duration of each session

Training duration included 50 min, 60 min, and 90 min. We divided these durations into three subgroups. In the comparison between subgroups, we found no significant difference between subgroups in the effect of the duration of each session on UPDRS-III scores (*p* = 0.6; I^2^ = 0%) for all tests between subgroups. However, a 60-min exercise session was the most common choice. A duration of 60 min of exercise per session had a significant effect (MD = -5.82; 95% CI, -8.75, -2.89). There were no differences among the subgroups in terms of TUG scores (*P* = 0.89; I^2^ = 0). A duration of 60 min of exercise per session had a significant effect (MD = -1.96; 95% CI, -2.79, -1.13) on TUG scores. The frequency of exercise per week affects motor symptoms and may also be related to the area of evaluation.

#### Duration of exercise per week

Because of the different training times, the total training time in a week was grouped. The weekly exercise duration included in this study was divided into 60 min, 120 min, 150 min, 180 min, and > 180 min subgroups. The difference between subgroups was not significant for UPDRS-III scores (*P* = 0.24, I^2^ = 29.1%) or TUG scores (*P* = 0.36, I^2^ = 6.3%). Between 120 and 180 min of total exercise time per week was the most selected parameter. We also analyzed the relationship between the total time in one week and efficacy in the subsequent meta-regression analysis.

#### Exercise types

**Fig. 6:**
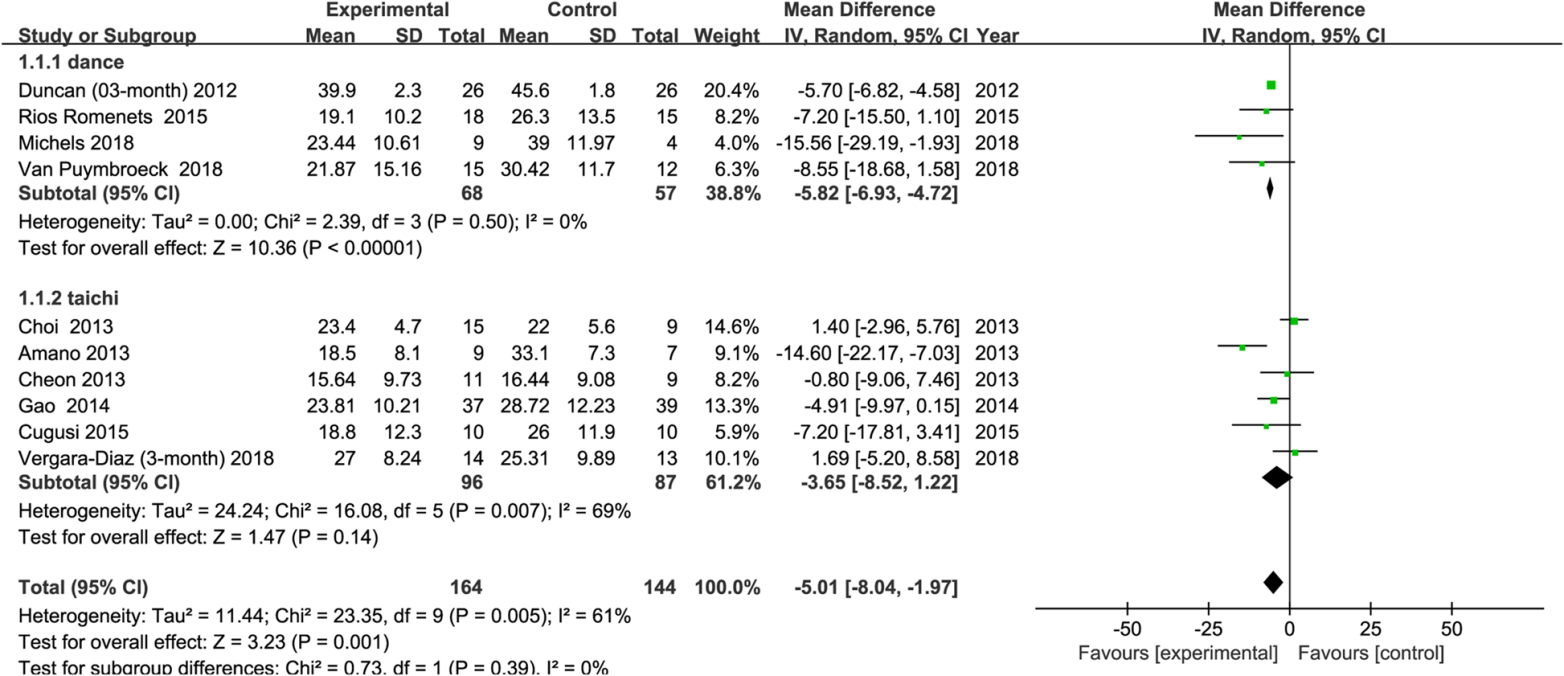
Funnel plot: Comparison of the tango and tai chi subgroups

For the primary outcome measure UPDRS-III scores, tango, and tai chi were the main forms of exercise. We selected studies with 2–3-month interventions and divided them into tango and tai chi subgroups, including 4 and 6 studies, respectively. Figure 6 shows that there was no significant difference in the subgroup analysis (*P* = 0.39, I^2^ = 0%).

### Meta-regression analysis

Meta-regression showed that the average age of participants affected the UPDRS-III scores (β = -0.423; 95% CI, -0.742, -0.106; *p* = 0.012). We also found that the duration of treatment affected the performance of the UPDRS (β = -0.121; 95% CI, -1.987, 0.043; *p* = 0.0005). When multivariate regression analysis was performed, the heterogeneity of age and treatment duration was reduced (Table 4).

**Table 4:** Meta-regression for UPDRS-III

	Univariate meta-analysis Coefficient (95% CI)	P	Multiple meta-regression Coefficient (95% CI)	P
Type of exercise	0.403(-0.492, 1.299)	0.354	0.115(-1.162, 1.393)	0.841
Area	-0.376( -0.654, 1.408)	0.450	-0.582( -2.430, 1.266)	0.489
Year	0.237( -0.031, 0.504)	0.079	0.276( -0.051, 0.604)	0.088
Disease stage	-1.763( -3.779, 0.251)	0.082	-2.752( -10.488, 4.983)	0.436
Age	-0.423 (-0.742, -0.106)	**0.012**	-0.210 (-0.840, 1.259)	0.657
Frequency of week	1.046(-0.436, 2.529)	0.154	31.425(-76.546, 13.695)	0.147
Time for session	-0.003(-0.122, 0.116)	0.955	-1.119(-2.676, 0.439)	0.136
Time for week	0.018(-0.007, 0.044)	0.146	0.553(-0.222, 1.328)	0.138
Duration of exercise	-0.121(-1.987, 0.043)	**0.005**	-0.188(-1.987, -0.000)	**0.050**

## Discussion

We systematically and comprehensively studied the effect of exercise on motor function and optimal exercise prescriptions among patients with PD. We found that many community-based exercises were an economical and convenient form of physical therapy and a continuation for PD patients after hospital treatment to maintain effects. The key findings of this study were as follows: 1) community-based exercise improved motor symptoms; (2) the most common exercise parameters were 60 min each session, twice a week, and tango and tai chi were the most common movement modalities; 3) the frequency of weekly exercise, age, and the duration of treatment of participants were important factors that affected the results; and 4) no significant heterogeneity was found in subgroup analysis or meta-regression analysis for tango, tai chi, or other exercise methods.

Heterogeneity was so ubiquitous that even if we performed a subgroup analysis or tried to exclude a single study, the heterogeneity could not be significantly changed. The higher heterogeneity is consistent with the results of other exercise interventions [58]. This suggests that the improvement of motor symptoms in PD by exercise is affected by multiple factors. The clinical manifestations of *PD* vary widely, even in motor symptoms [59]. PD is not just a motor disease; there are many nonmotor symptoms, such as salivary gland issues, visual disturbances, constipation, anxiety, and depression [4, 59]. These nonmotor symptoms can also lead to a reduced willingness to exercise among people with PD. People with PD often fall, making them sedentary and reluctant to exercise [60]. Complications other than Parkinson's motor disease may affect treatment outcomes. The incidence is higher among men than among women at every age [61], but women have a higher mortality rate and progress faster [62, 63]. The optimal parameters for exercise are different for men and women. Sex is also one of the factors that affects exercise among people with PD. The proportion of women is also a factor in the effect. There is still heterogeneity in the same forms of exercise, such as tai chi, dance, and tango groups. However, the heterogeneity between different exercise groups was not obvious. Therefore, our Parkinson's patients do not need to be confused about the optimal form of exercise, and the effects of different exercise forms may be slightly different. This is good news for people who cannot visit a rehabilitation center to see a physical therapist because of financial or transportation reasons. PD is a progressive disease of the nervous system, which may be the reason why age is an important factor. The duration of treatment should be studied for three or six months in the future to increase the homogeneity of the study. If the treatment course is too short, the patient may learn various motor skills, which may exaggerate the curative effect; if it is too long, since PD is a progressive disease [30], it will not be comparable to other treatment cycles. Long-term exercise has not been found to have adverse results among older adults and PD patients [26, 64]. For patients, weekly and lifelong training is recommended. A more standardized, rigorous scientific design is also needed to explore the best exercise prescription.

Our study also has some shortcomings: 1) due to the large heterogeneity, we did not conduct evidence-level classification; 2) we were unable to include studies with the same movement parameters, and more studies on parameters and movement forms are needed in the future; 3) we did not account for changes in other parameters in the subgroup analysis; and 4) some studies did not report the number of men and women, and the sex ratio was not considered as one of the multiple factors in the meta-regression analysis.

It is important to note that in 2020, the WHO recommended 150–300 min of moderate-intensity physical activity per week for all adults, for people over 65 years of age, and for people with disabilities [65]. PD mostly affects people over 60 years of age, and the age at onset of the disease is usually 65 to 70 years [66]. A study in northern Sweden showed that the average age of people with PD was 70.6 years [67]. Moderate-intensity training was beneficial for all older adults over the age of 85, and there was no significant increase in adverse events [68]. Studies have shown no difference in 5-year mortality between moderate-intensity and high-intensity exercise interventions but a downward trend compared with the control group [69]. Studies have shown that exercise is safe and effective in reversing functional decline caused by acute hospitalization among elderly patients (mean age: 87.3; range, 75–101) [70]. Therefore, we believe that the WHO recommendations also apply to PD patients. People with PD have reduced activities of daily living and exercise less per week than healthy elderly people. Studies have shown that intensive exercise therapy can be beneficial for PD patients [71]. Surveys show a global trend of inactivity [72, 73]. People with PD may not have performed exercise that was intense enough in the current study. We should appropriately increase the exercise time of patients in future studies.

## Conclusions

This meta-analysis suggests that community-based exercise may benefit motor function in patients with PD. This provides strong evidence for patients to choose more rehabilitation pathways.

The most commonly used modalities of exercise were tango and tai chi, and the most common prescription was 60 min twice a week. However, more direct studies comparing these parameters should be conducted in the future. Age, duration of treatment, and weekly frequency were important factors that influenced the effect.

## Supplementary Information

**Additional file 1. **

## Data Availability

All data generated or analyzed in this work are included in the published version of the manuscript.

## Abbreviations

PD: Parkinson’s disease
UPDRS-III: Unified Parkinson’s Disease Rating Scale part III
MD: Mean difference
TUG: Timed Up and Go test
UPDRS: Unified Parkinson’s Disease Rating Scale
BBS: Berg Balance Scale
6MWT: 6-Minute Walk Test
H&Y: Hoehn and Yahr
