# Supplementary material for: Effects and parameters of community-based exercise on motor symptoms in Parkinson’s disease: a meta-analysis
Source: BMC Neurol. 2022 Dec 29;22:505. doi: 10.1186/s12883-022-03027-z (PMC9797903; doi:10.1186/s12883-022-03027-z)
Supplement: Supplementary file 1 — Additional file 1. [file 12883_2022_3027_MOESM1_ESM.docx]

| PubMed | Search Strategy |
| --- | --- |
| #1 | (((((((((((Parkinson Disease[MeSH Terms]) OR (Idiopathic Parkinson's Disease[Title/Abstract])) OR (Lewy Body Parkinson's Disease[Title/Abstract])) OR (Parkinson's Disease, Idiopathic[Title/Abstract])) OR (Parkinson's Disease, Lewy Body[Title/Abstract])) OR (Parkinson Disease, Idiopathic[Title/Abstract])) OR (Parkinson's Disease[Title/Abstract])) OR (Idiopathic Parkinson Disease[Title/Abstract])) OR (Lewy Body Parkinson Disease[Title/Abstract])) OR (Primary Parkinsonism[Title/Abstract])) OR (Parkinsonism, Primary[Title/Abstract])) OR (Paralysis Agitans[Title/Abstract]) |
| #2 | (((((((((((((((((((((((((Exercise[MeSH Terms]) OR (Exercises[Title/Abstract])) OR (Physical Activity[Title/Abstract])) OR (Activities, Physical[Title/Abstract])) OR (Activity, Physical[Title/Abstract])) OR (Physical Activities[Title/Abstract])) OR (Exercise, Physical[Title/Abstract])) OR (Exercises, Physical[Title/Abstract])) OR (Physical Exercise[Title/Abstract])) OR (Physical Exercises[Title/Abstract])) OR (Acute Exercise[Title/Abstract])) OR (Acute Exercises[Title/Abstract])) OR (Exercise, Acute[Title/Abstract])) OR (Exercises, Acute[Title/Abstract])) OR (Exercise, Isometric[Title/Abstract])) OR (Exercises, Isometric[Title/Abstract])) OR (Isometric Exercises[Title/Abstract])) OR (Isometric Exercises[Title/Abstract])) OR (Exercise, Aerobic[Title/Abstract])) OR (Aerobic Exercise[Title/Abstract])) OR (Aerobic Exercises[Title/Abstract])) OR (Exercises, Aerobic[Title/Abstract])) OR (Exercise Training[Title/Abstract])) OR (Exercise Trainings[Title/Abstract])) OR (Training, Exercise[Title/Abstract])) OR (Trainings, Exercise[Title/Abstract]) |
| #3 | (((Sports[MeSH Terms]) OR (Sport[Title/Abstract])) OR (Athletics[Title/Abstract])) OR (Athletic[Title/Abstract]) |
| #4 | (rehabilitation[MeSH Terms]) OR (Habilitation[Title/Abstract]) |
| #5 | ((((((((((((((((((Physical Therapy Modalities[MeSH Terms]) OR (Modalities, Physical Therapy[Title/Abstract])) OR (Modality, Physical Therapy[Title/Abstract])) OR (Physical Therapy Modality[Title/Abstract])) OR (Physiotherapy (Techniques[Title/Abstract]))) OR (Physiotherapies (Techniques[Title/Abstract]))) OR (Physical Therapy Techniques[Title/Abstract])) OR (Physical Therapy Technique[Title/Abstract])) OR (Techniques, Physical Therapy[Title/Abstract])) OR (Group Physiotherapy[Title/Abstract])) OR (Group Physiotherapies[Title/Abstract])) OR (Physiotherapies, Group[Title/Abstract])) OR (Physiotherapy, Group[Title/Abstract])) OR (Physical Therapy[Title/Abstract])) OR (Physical Therapies[Title/Abstract])) OR (Therapy, Physical[Title/Abstract])) OR (Neurological Physiotherapy[Title/Abstract])) OR (Physiotherapy, Neurological[Title/Abstract])) OR (Neurophysiotherapy[Title/Abstract]) |
| #6 | (((#2) OR (#3)) OR (#4)) OR (#5) |
| #7 | (#1) AND (#6) |
| #8 | (#1) AND (#6) Filters: Clinical Trial, Randomized Controlled Trial |

| PEDro | Search Strategy |
| --- | --- |
|  | Parkinson disease [Abstract & Title] Filters: Clinical Trial[method] |

|  | |
| --- | --- |
| CENTRAL | Search Strategy |
| #1 | Parkinson’s Disease [MeSH Terms] |
| #2 | Exercise [MeSH Terms] |
| #3 | Sports [MeSH Terms] |
| #4 | Rehabilitation [MeSH Terms] |
| #5 | Physical Therapy Modalities [MeSH Terms] |
| #6 | (((#2) OR (#3)) OR (#4)) OR (#5) |
| #7 | (#1) AND (#6) in trials |


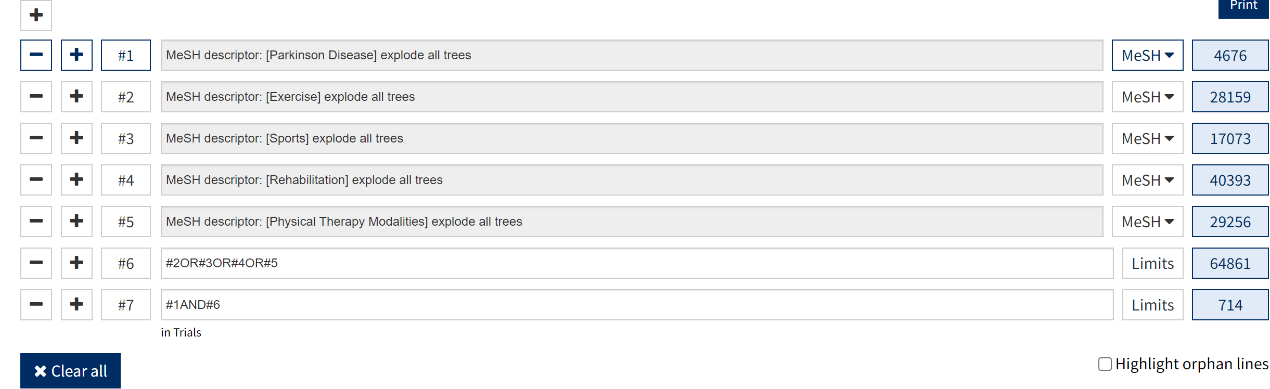


| Embase | Search Strategy |
| --- | --- |
| #1 | **'exercise'**/exp |
| #2 | **'sport'**/exp |
| #3 | **'rehabilitation'**/exp |
| #4 | **'physiotherapy'**/exp |
| #5 | #1 OR #2 OR #3 OR #4 |
| #6 | **'Parkinson disease'**/exp |
| #7 | #5 AND #6 |
| #8 | **#5 AND #6 AND ([controlled clinical trial]/lim OR [randomized controlled trial]/lim) AND [english]/lim** |

| Scopus | Search Strategy |
| --- | --- |
|  | (TITLE-ABS-KEY (exercise) R TITLE-ABS-KEY (sports) OR TITLE-ABS-KEY (rehabilitation) OR TITLE-ABS-KEY (physiotherapy)) AND TITLE-ABS-KEY (Parkinson AND disease )  AND  ( LIMIT-TO ( DOCTYPE,  "ar" ) )  AND  ( LIMIT-TO ( LANGUAGE,  "English" ) )  AND  ( LIMIT-TO ( SUBJAREA,  "MEDI" )  OR  LIMIT-TO ( SUBJAREA,  "NEUR" )  OR  LIMIT-TO ( SUBJAREA,  "HEAL" )  OR  LIMIT-TO ( SUBJAREA,  "BIOC" )  OR  LIMIT-TO ( SUBJAREA,  "PSYC" ) )  AND  ( LIMIT-TO ( SRCTYPE,  "j" ) ) |

| WOS | Search Strategy |
| --- | --- |
|  | ((TS=(exercise) OR TS=(sports) OR TS=(rehabilitation) OR TS=(physiotherapy))) AND TS= (Parkinson disease)  Refined By: Document Types: Articles. Languages: English |
